# A concept analysis of *‘trial recruitment’ *using the hybrid model – Phase 1 findings

**DOI:** 10.12688/hrbopenres.13173.1

**Published:** 2020-12-21

**Authors:** Hannah Delaney, Declan Devane, Andrew Hunter, Shaun Treweek, Nicola Mills, Carrol Gamble, Valerie Smith

**Affiliations:** 1 Health Research Board - Trials Methodology Research Network, National University of Ireland, Galway, Galway, Ireland; 2 School of Nursing and Midwifery, National University of Ireland, Galway, Galway, Ireland; 3 School of Nursing and Midwifery, Trinity College Dublin, Dublin, Ireland; 4 Qualitative Research in Trials Centre (QUESTS), National University of Ireland, Galway, Galway, Ireland; 5 Health Services Research Unit, University of Aberdeen, Aberdeen, UK; 6 Population Health Sciences, Bristol Medical School, University of Bristol, Bristol, UK; 7 Clinical Trials Research Centre, University of Liverpool, Liverpool, UK

**Keywords:** Concept analysis, Trial recruitment, Trial report, Trial enrolment

## Abstract

**Background: **The International Committee of Medical Journal Editors (ICMJE) requires trials submitted for publication to be registered before enrolment of the first participant; however, there is ambiguity around the definition of recruitment and in anchoring the trial start date, end date, recruitment and enrolment, temporally to trial processes. There is potential for variation in how recruitment is reported and understood in trial protocols and trial reports. We report on Phase 1 of a concept analysis of ‘trial recruitment’ and develop a preliminary operational definition of ‘trial recruitment’.

**Methods: **A concept analysis using the hybrid model. We searched randomised and non-randomised trial reports published between January 2018 and June 2019. Included studies were sourced from the five top journals in the category of medicine with the highest impact factor. We examined how recruitment was defined temporally to four time points; screening, consent, randomisation, and allocation.

**Results: **Of the 150 trial reports analysed, over half did not identify a clear time point of when recruitment took place in relation to any of screening/consent/randomisation/allocation. The majority of the assessed trials provided a time frame in relation to the trial (i.e. start/end date), the process that this time frame referred to differed between studies. There was variation across studies in the terminology used to describe entry to the trial and often multiple terms were used interchangeably.

**Conclusion: **There is ambiguity around temporal descriptions of ‘trial recruitment’ in health care journals. Informed by the findings of Phase 1, we developed a preliminary temporal operational definition of trial recruitment based on i) trial recruitment of an individual or cluster and ii) the trial recruitment period. In Phase 2 this definition will be discussed in focus groups with healthcare workers involved in designing/implementing/reporting on trials; to contribute to the final phase (analytical phase) of this concept analysis.

## Background

Non-reporting of completed trials and selective outcome reporting in trials can result in a biased assessment of the global body of evidence that inform health care decisions. Since 2004, the International Committee of Medical Journal Editors (ICMJE) has required that trials submitted for publication must be registered before the enrolment of the first trial participant. The ICJME ‘ *does not define the timing of first participant enrolment, but best practice dictates registration by the time of first participant consent’* ^1^ (p. e1). In addition, the World Health Organisation’s (WHO) ‘International Standards for Clinical Trial Registries’ ^2^ define prospective trial registration as *‘the registration of a trial before the recruitment of the first participant’* (p.8) and date of the first enrolment as the *‘anticipated or actual date of enrolment of the first participant’* (p.28) but the temporal relationship between an invitation to potential participant, consent and randomisation is not defined. For example, the International Standard Randomised Controlled Trial Number (ISRCTN) registry defines a recruitment start date as *‘the date, or planned date, of recruitment of the first participant to the study’* ^3^ (p. e1). The clincialtrials.gov registry refers to a study start date and defines this as ‘ *the estimated date on which the clinical study will be open for recruitment of participants, or the actual date on which the first participant was enrolled’* ^4^ (p. e1), thus separating recruitment from enrolment whereby a participant is ‘enrolled’ following completion of the informed consent process.

In summary, ambiguity in anchoring the trial start date, end date, recruitment and enrolment temporally to trial processes (e.g. invitation, consent, and randomisation) has the potential for variation in how recruitment is reported and understood in trial registries, trial protocols and trial reports.

## Aim

To report Phase 1 of a concept analysis of ‘trial recruitment’ using the hybrid model ^5^. The aim of Phase 1 is to develop a preliminary operational definition of ‘trial recruitment’, which is then further explored and refined following Phases 2 and 3.

## Methods

### Study design

**Figure 1.**
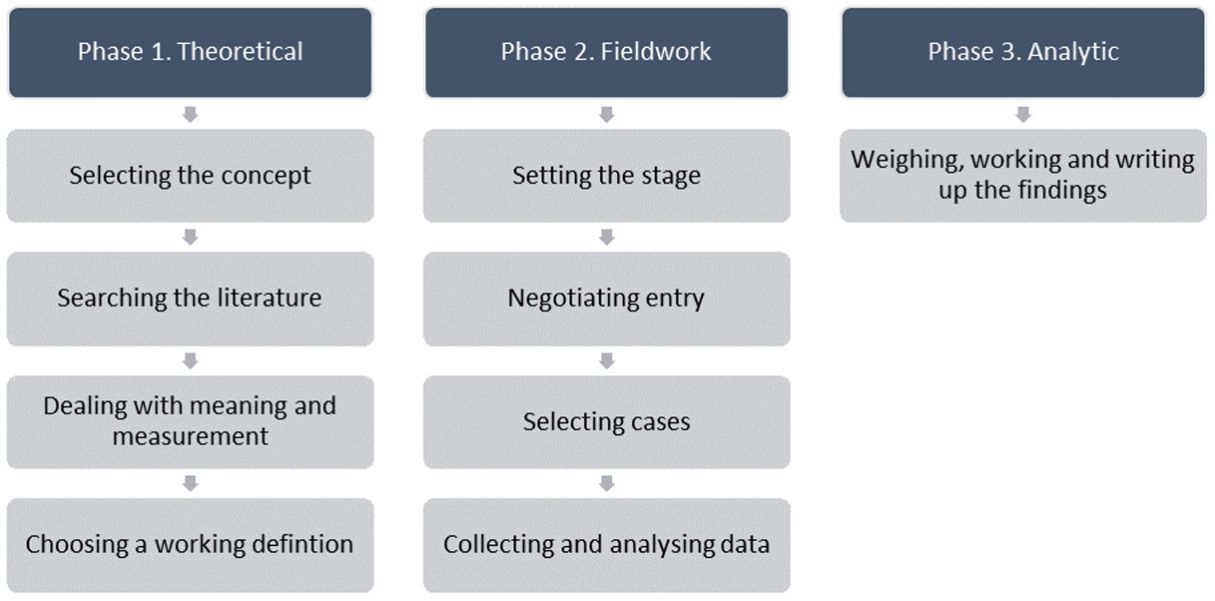
Phases of the hybrid model of concept analysis ^5^.

A concept analysis typically involves synthesising evidence on a concept and distinguishing it from other similar/related concepts to help resolve inconsistencies in the knowledge base ^6^. Concept analysis offers a means of defining or clarifying concepts, contextually, while also assisting to elucidate patterns of usage which can become a precursor of theory and knowledge development ^7^. There are various methods available for formal concept analyses ^8^. We chose Schwartz-Barcott and Kim’s hybrid model to analyse the concept of ‘trial recruitment’, because it is considered beneficial in helping resolve ambiguity surrounding a concept and is facilitative of concept expansion and purification ^9^. The model consists of three major phases; 1) the theoretical phase, 2) the fieldwork phase, and 3) the analytic phase ( Figure 1). This paper reports on Phase 1.

### Searching the literature

The theoretical phase aims to comprehensively source and analyse relevant literature to acquire a deep understanding of the concept under study; that is, how the concept has been defined, used, and ways that it has been or might be measured ^5^. To gain a contemporary understanding of the concept of ‘trial recruitment,’ we searched randomised (parallel, cluster, and other randomised designs, including pilot and feasibility trials) and non-randomised (i.e. quasi) trial reports published between January 2018 and June 2019. Included studies were sourced from the five top journals in the category of medicine ^10^ that had the highest impact factor ( Table 1). We excluded trial protocols, studies reporting secondary analyses of original/primary trial data, trials not yet started, ongoing studies, meta-analyses/systematic reviews and single-arm studies.

**Table 1.** Top five impact factor medical journals 2019 ^10^.

Journal Title	Impact factor (2019)
New England Journal of Medicine (N Engl J Med)	55.873
Lancet	45.217
Journal of the American Medical Association (JAMA)	35.289
Annals of Internal Medicine (Ann Intern Med)	17.81
British Medical Journal (BMJ)	17.445

The search strategy (available as *Extended data* ^11^) was executed in June 2019, using the Cochrane Collaboration’s EMBASE ‘trial’ search string ^12^ combined with the respective journal titles, and limited by year 2018-2019 and ‘article’ publication type.

### Dealing with meaning and measurement

The following data were extracted and used to analyse the concept of *‘trial recruitment’*; study characteristics (data source, the aim of the study, location of study, and health condition); implicit or explicit temporal descriptions and definitions of the trial start date, end date, trial duration, gaining consent, recruitment, enrolment, and randomisation. Once data were extracted, significant points of contrast and similarity were explored. This type of comparison gives the researcher an insight into the degree of consensus among users of the concept of ‘trial recruitment’ and can help ascertain the degree of intersubjectivity of meaning ^5^. Anticipating that few explicit definitions of trial recruitment might exist, Schwartz-Barcott and Kim recommend analysis of the authors’ writings to determine implied definitions of the concept under study, using the format given in Table 2 as a guide ^5^.

**Table 2.** Sample format for organising and analysing definitions ^5^.

*Reference*	*Explicit*	*Implicit*	*Examples*	*Comments*
				

### Data analysis

**Figure 2.**
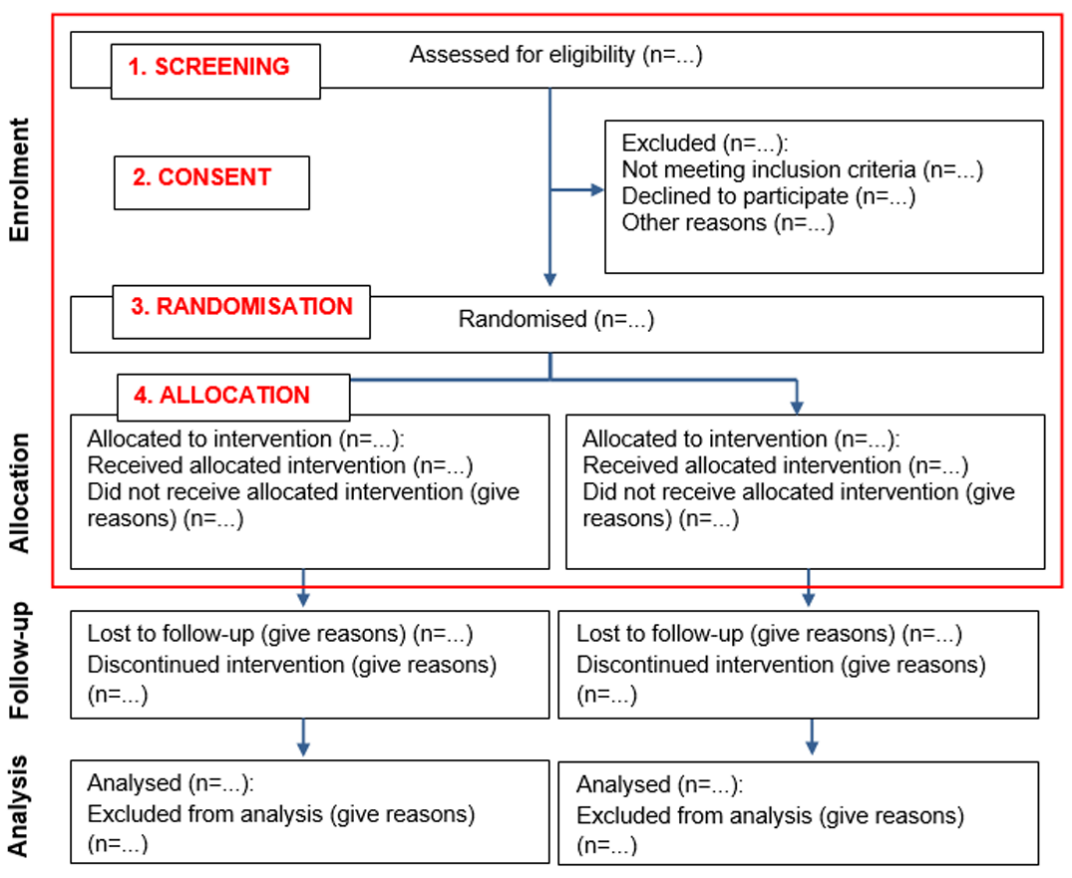
CONSORT flow diagram ^13^ - edited to include the four time points analysed for this study.

The CONSORT flow diagram ^13^ recommends that five main time points should be reported when presenting the progress of participants through a trial (enrolment, randomisation, allocation, follow-up and analysis). As we were concerned explicitly with recruitment in this analysis, we focused on enrolment, randomisation and allocation. For our analysis, we examined how recruitment was defined temporally to four time points, these are aligned with the CONSORT time points; 1. screening (i.e. CONSORT enrolment/eligibility assessment), 2. consent (i.e. CONSORT enrolment/exclusion), 3. randomisation and 4. allocation (see Figure 2).

## Findings of Phase 1

### Results of the search

**Figure 3.**
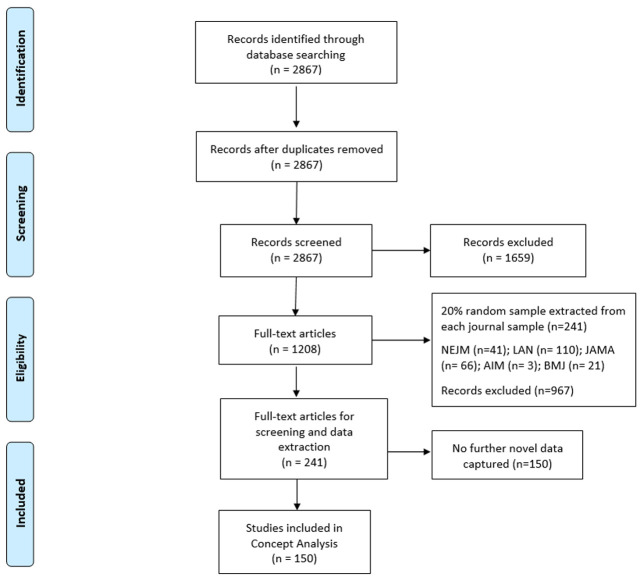
PRISMA flow diagram ^14^

Searches yielded 2867 records, and no duplicates were found. Following title and abstract screening, 1659 records were excluded based on our predefined inclusion and exclusion criteria. Given the scope of our inclusion criteria, we were confident that the majority of the 1208 records would be included following full-text screening. For this reason, we decided to do full-text screening and data extraction concurrently, dividing the papers equally between three authors (HD, VS and AH). After piloting the data extraction form with a subset of 10 of the 1208 records, and considering the similar reporting format across the five included journals, we selected a 20% random sample of records from each of the five journals, resulting in the inclusion of 241 studies on which to base the concept analysis (see *Extended data* ^11^). Although we anticipated extracting data from all 241 included studies, at 150 records we had reached a point where no further novel data were being captured (see Figure 3 for further details). For this reason, we concluded data extraction with these 150 trial reports and based the theoretical analysis on the data extracted from these 150 records as we believed this offered data sufficiency in meeting the aim of Phase 1 of this concept analysis. We recognise, however, in omitting the additional 91 records, that the proportions reported in our findings may have been impacted on but not necessarily on the overall conclusions derived from the analysis. 148 of the records reported on randomised trials and two reported on non-randomised trials.

### Characteristics of included studies

Of the included trials, 21 reported on trials in oncology, with the remaining trials reporting in the medical areas of: cardiology (n=6), psychiatry (n=6), diabetes (n=5), stroke (n=5), dermatology (n=5), HIV (n=5), paediatrics (n=4), ophthalmology (n=4), and other (n=89) (see *Extended data* ^11^). The majority of trials were carried out in multiple countries (n=55). Twenty-nine were based in America and 15 in the United Kingdom. The remaining trials were conducted in: Asia (n=9), Australia (n=7), Africa (n=5), Netherlands (n=5), France (n=5), Germany (n=4), Switzerland (n=3), Norway (n=2), Canada (n=2), not stated (n=3), and one in each of Hong Kong, Ireland, Poland, Portugal, Russia, South Africa.

### Temporal descriptions of ‘recruitment’

**Figure 4.**
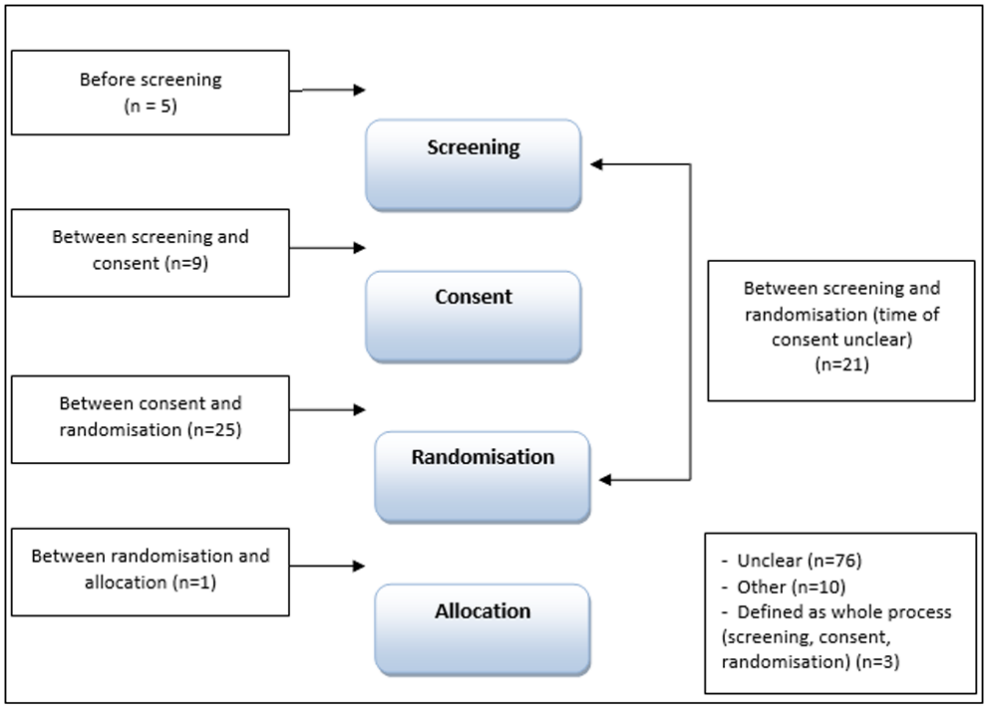
At what time point is recruitment defined?

Of the 150 trials analysed, over half (n=76) did not identify a clear time point of when recruitment took place in relation to any of screening, consent, randomisation, allocation (see Figure 4). Twenty-five of the trial reports referred to recruitment as taking place *after consent and before randomisation* (explicit n=15, implicit n=10); 21 as the point *between screening and randomisation* (explicit n=10, implicit n=11) with the timing of consent unspecified; and nine referred to recruitment as the point *between screening and consent* (explicit n=3, implicit n=6). The remaining trials defined recruitment at the time-point *before screening* (n=5, 3 explicit and 2 implicit); *between randomisation and allocation* (n=1, explicit). Three studies referred to recruitment generally as including screening, consent and randomisation (explicit n=1, implicit n=2), 10 were categorised as ‘other’: in seven of these trial reports the order of trial processes differed to the order identified in the CONSORT flow diagram and three trials referred to recruitment taking place at randomisation, but the timing of randomisation was unclear.

The majority of the assessed trials (n=138) provided a time frame in relation to the trial (i.e. start and end date); however, the process that this time frame referred to differed between studies (see Table 3). For instance, 24 studies included the start and end date of the duration of the trial such as ^15^; *'...multicentre phase 3 trial was conducted from August 4, 2011, to June 20, 2017’* (p.599). Twenty-two studies stated the start and end date of the randomisation period, such as ^16^; *'Between Oct 1, 2012, and June 20, 2014, we randomly assigned 155 participants...'* (p.41). Others included dates between which ‘enrolment’ (n=18), ‘recruitment’ (n=15), and ‘screening’ (n=13) took place. Forty of the trials reported on the start and end date of multiple processes, for instance:

**Table 3.** Reported start and end date.

Reported on the start and end date for:	Total studies
Multiple recruitment processes (i.e. the time frame provided referred to more than one process, such as ‘enrolment and randomisation’)	40
Trial duration (i.e. providing a start and end date for the ‘study’ or ‘trial’ period)	24
Period of randomisation (i.e. reporting the start and end date for when ‘randomisation’ took place)	22
Period of enrolment (i.e. reporting the start and end date for when ‘enrolment’ took place)	18
Period of recruitment (i.e. reporting the start and end date for when ‘recruitment’ took place)	15
No start/end date reported	12
Screening period (i.e. reporting the start and end date for when ‘screening’ took place)	13
Other (i.e. reporting a time frame for trial processes not related to ‘recruitment’ such as data collection and rounds of treatment)	6
Total	150

*‘During the study period (August 2015 and May 2017), 151 patients were screened, 117 underwent randomization’* ^17^ (p.2301)
*‘Between July 2, 2013, and May 10, 2016, 80 patients were enrolled, randomly assigned, and started their allocated treatment'* ^18^ (p.328)

The studies categorised as ‘other’ (n=6) reported on the start and end date of other processes such as data collection (n=1), rounds of treatment (n=2), and the use of the same start and end date with differing terminology (n=3); for instance, enrolment and recruitment were used interchangeably. Further findings on the variation in language are presented in the next section.

### Variation in terminology

There was variation across the studies in the terminology used to describe entry (the point at which a participant was considered to have ‘joined’ a trial) to the trial (see Table 4). Of the 150 analysed trials, just over a third (n=52) used the term ‘enrolment’, and 34 did not use a specific term to describe entry to the trial. Thirty studies used multiple terms; this was mostly in the form of ‘recruitment’ used interchangeably with another term such as ‘enrolment’ (n=19), ‘randomisation’ (n=3), randomisation and enrolment (n=2), screening (n=1), screening and randomisation (n=1). Other studies used the term ‘randomisation’ interchangeably with ‘accrued’ (n=1) and ‘enrolment’ (n=1). One study used the terms ‘included’ and ‘enrolment’ interchangeably. Table 4 and Table 5 illustrate the variation across the studies in the terminology used to describe the entry of participants to the trial.

**Table 4.** Terminology.

Term used to describe entry to the trial	Total studies
Enrolment	52
No specific term used	34
Multiple terms used	30
Recruitment	29
Other	5
Total	150

**Table 5.** Variation in terminology.

*Journal [reference]*	*Healthcare area*	*Description*
N Engl J Med ^19^	Lung Disease	*‘863 infants were enrolled during the period from April 2010 through August 2013’*(p.149)
Lancet ^20^	Osteoperosis	*‘...we enrolled post-menopausal women with at least two moderate or one severe* *vertebral fracture and a bone mineral density’* (p.30) *‘We enrolled 680 patients in each group…’* (p.30)
Lancet ^21^	Oncology	*‘Of 601 patients assessed for eligibility, a total of 452 patients… were recruited and* *randomly assigned’* (p.233) *‘...601 patients assessed for eligibility, of whom 452 patients were enrolled and 226 were* *randomly assigned ’* (p.229)
JAMA ^22^	Anaesthesia	*'Patients undergoing anaesthesia with RSI were enrolled from February 2014 until* *February 2017...'* (p.E1) *'... Recruitment began in February 2014 and ended in February 2017’* (p.E2)
Lancet ^23^	Adolescent health	*‘Of the 112 eligible schools, 75 were randomly selected to participate in the trial...' (p.2471)* *'...we recruited 75 schools'* (p.2471)
JAMA ^24^	Retinopathy of prematurity	*‘Patients were recruited between September 2014 and August 2016. 20 infants were* *screened and 19 were randomized’…(p.278)* *‘20 patients were screened and 19 were enrolled'* (p.279)
Lancet ^25^	Inflammatory diseases	*‘Between Oct 6, 2015, and Nov 30, 2016, 166 patients were screened, of whom 102 were* *randomly assigned ...’* (p.1330) *‘...Patients were recruited between Oct 6, 2015, and Nov 30, 2016’* (p.1335)
BMJ Open ^26^	Critical care	*‘...an enrolment of 114 patients was planned....’* (p.3) *‘One hundred fourteen patients were included in this study with 57 patients randomised in* *each group’* (p.3)

## Conclusion and working definition for Phase 2

The theoretical Phase 1 of our concept analysis has revealed that there is ambiguity around temporal descriptions of ‘trial recruitment’ in health care journals, and varying terminology is used when reporting on trial recruitment.

Sixty-one of the analysed trials identified a time point, in relation to the four main trial processes (screening, consent, randomisation, allocation), at which trial recruitment took place. The majority of these studies identified trial recruitment as being between consent and randomisation or between screening and randomisation (with time of consent unclear) as the time point of actual recruitment. Over half of the trials analysed (n=76) did not identify a clear time point of when trial recruitment took place. Our analysis also revealed a variation in terminology used to describe entry to the trial, and often multiple terms were used interchangeably. Enrolment (n=52) and recruitment (n=29) were the most common terms used, but the use of numerous terms was also frequent in the trial reports (n=30).

There are some limitations of this study to be noted. We acknowledge that trial design could potentially impact on the variation and type of terminology used when reporting trials, for instance whether or not a trial is randomised and whether the trial includes a run-in period. We did not extract data relating to trial run-in periods and the majority of the trials analysed here are randomised trials. However, we included both randomised and non-randomised trials in our search strategy and the selection of trials for inclusion in analysis was based on a random sample of records from each of the five journals.

Considering these findings, we have developed a preliminary temporal operational definition of trial recruitment based on i) trial recruitment of an individual or cluster as ‘the time point after screening and consent and before randomisation’ and ii) trial recruitment period as ‘the time point after screening and consent of the first participant, and before randomisation of the last participant’. According to Schwartz-Barcott and Kim ^5^, once an initial definition is developed from the findings of the theoretical phase of the analysis, a ‘further detailed examination’ of the definition is required. This occurs in Phase 2 during fieldwork. Our definition from Phase 1 will be discussed in focus groups with healthcare workers involved in designing, implementing and reporting on trials. Further discussion around temporal descriptions and reporting of ‘trial recruitment’ will take place during these focus groups. The use of varying terminology when reporting on trial recruitment will also be further explored in the focus group discussions. The findings from the focus groups will be combined with the findings as above, in the final phase of this concept analysis process; Phase 3, the analytical phase.

## Data availability

### Underlying data

Figshare: https://doi.org/10.6084/m9.figshare.13109870.v1 ^11^

This project contains the following underlying data:

- Delaney *et al.* 2020_Concept Analysis_Extracted Data.xlsx

### Extended data

Figshare: https://doi.org/10.6084/m9.figshare.13109870.v1 ^11^

This project contains the following extended data:

- Delaney *et al.* 2020_search strategy.pdf
- Delaney *et al.* 2020_records per journal.pdf
- Delaney *et al.* 2020_characteristics of included studies.pdf

Data are available under the terms of the Creative Commons Attribution 4.0 International license (CC-BY 4.0).
