# Supplementary material for: A concept analysis of ‘trial recruitment’ using the hybrid model
Source: HRB Open Res. 2022 Apr 1;3:92. Originally published 2020 Dec 21. [Version 2] doi: 10.12688/hrbopenres.13173.2 (PMC9021666; doi:10.12688/hrbopenres.13173.2)
Supplement: Peer review history

# Peer review: Reviewer response for version 2

**DOI:** 10.21956/hrbopenres.14754.r31776

**Published:** 2022-04-13

**Authors:** Jennifer Hanratty

**Affiliations:** 1 Centre for Evidence and Social Innovation, Queen's University Belfast, Belfast, UK; 2 Campbell UK & Ireland, Queen's University Belfast, Belfast, UK

Thank you for your additions and clarifications.

Is the work clearly and accurately presented and does it cite the current literature?

Yes

If applicable, is the statistical analysis and its interpretation appropriate?

Not applicable

Are all the source data underlying the results available to ensure full reproducibility?

Yes

Is the study design appropriate and is the work technically sound?

Yes

Are the conclusions drawn adequately supported by the results?

Partly

Are sufficient details of methods and analysis provided to allow replication by others?

Yes

Reviewer Expertise:

Systematic reviews and evidence synthesis, evaluation (including trials).

I confirm that I have read this submission and believe that I have an appropriate level of expertise to confirm that it is of an acceptable scientific standard.

# Peer review: Reviewer response for version 2

**DOI:** 10.21956/hrbopenres.14754.r31775

**Published:** 2022-04-01

**Authors:** Hans Lund

**Affiliations:** 1 Section for Evidence-Based Practice, Western Norway University of Applied Sciences, Bergen, Norway

No further comments.

Is the work clearly and accurately presented and does it cite the current literature?

Partly

If applicable, is the statistical analysis and its interpretation appropriate?

Yes

Are all the source data underlying the results available to ensure full reproducibility?

Yes

Is the study design appropriate and is the work technically sound?

Yes

Are the conclusions drawn adequately supported by the results?

Yes

Are sufficient details of methods and analysis provided to allow replication by others?

Yes

Reviewer Expertise:

Rehabilitation - Systematic Reviews - Evidence-Based Medicine - Evidence-Based Research

I confirm that I have read this submission and believe that I have an appropriate level of expertise to confirm that it is of an acceptable scientific standard.

# Peer review: Reviewer response for version 1

**DOI:** 10.21956/hrbopenres.14299.r28734

**Published:** 2021-02-09

**Authors:** Jennifer Hanratty

**Affiliations:** 1 Centre for Evidence and Social Innovation, Queen's University Belfast, Belfast, UK; 2 Campbell UK & Ireland, Queen's University Belfast, Belfast, UK

As a systematic review expert with experience of evaluating and conducting trials I am very well qualified to review this paper and I thank the authors for an interesting read.

 This study analysed reporting of trial recruitment timing in leading medical journals and found that reporting is unclear, incomplete or absent. This work is a timely reminder that incomplete or unclear reporting in RCTs is an issue that requires remedy. I commend the team for thoroughly documenting the problem.

**Abstract:** the team uses both "enrolment" and "recruitment" interchangeably in the abstract (and the background section). Is this deliberate or should it be revised to consistently use "recruitment", or perhaps offer some explication of the difference between the two terms (if any)?

**Background:** This situates the work well and cites relevant and current guidance. I would like to see a statement and some elaboration on why " *variation in how recruitment is reported and understood"* is a problem – aside from being a major frustration for systematic reviewers. The opening line appropriately mentions bias due to non-reporting or selective reporting but this is not directly linked to reporting on recruitment. I’d really like the authors to explicitly answer the questions; why is this a problem? What are the implications of poor reporting or indeed poor practice – why does it matter?

**Methods:** This is very clearly presented and I found the succinct introduction to concept analysis particularly informative. I am curious as to why trial protocols were excluded as they are a vital part of the reporting and often contain supplementary detail not contained in the trial report. Could the authors explain why protocols were excluded? I would be interested to know too if authors consulted the trial protocol of included published trials to get more information on reporting of recruitment and to compare reporting between protocol and publication.

 The selection of a subsample of trials is appropriate and well documented. In the PRISMA Flow diagram, box " *no further novel data captured"* – should this be 91 rather than 150?

 The line " *148 of the records reported on randomised trials and two reported on non-randomised trials."* would fit better in Characteristics of included studies. I would also like to know what proportion were cluster, parallel, pragmatic RCTs and if any were feasibility studies or pilot trials to get a fuller sense of the data set. Is it possible to explore whether or not the definition of recruitment was related to the type of trial?

 I suggest a change of wording " *Of the 150 trials analysed, over half (n=76) did not identify a clear time point of when recruitment took place in relation to any of screening, consent, randomisation, allocation"* to " *Of the 150 trials analysed, over half (n=76) did not clearly identify when recruitment took place in relation to any of screening, consent, randomisation, allocation".*

 I prefer reporting to be consistent in referring to proportions, percent or number and not mixing different metrics e.g., " *Of the 150 analysed trials, just over a third (n=52) used the term ‘enrolment’, and 34 did not use a specific term to describe entry to the trial."* Could be " *Of the 150 analysed trials, just over a third (n=52) used the term ‘enrolment’, and just over one fifth (n=34) did not use a specific term to describe entry to the trial."*

**Conclusions:** The lack of comparison between RCTs and non-RCTs is unfortunate but unsurprising given the focus on medical journals. A better approach may have been to focus only on randomised trials *or *to specifically search for non-RCTs, which would likely mean expanding the search to include non-medical journals/databases to capture non-RCTs as they are less common in medicine than other areas of health and social care.

 I understand that the focus is on the term "trial recruitment" but could you also consider that the term "enrolment" might have its own distinct meaning and explore this in the next phase of the study? Given that the term "enrolment" was actually more common in your data set, had you considered that enrolment might be the more useful term *instead *of recruitment or do you think they are two distinct terms with distinct meanings? The preliminary definition is very useful.

 As above, I would like a see the authors draw out the importance of good clear reporting, why terminology is important, and preliminary recommendations for how to fix the problem – not just providing a definition but telling the reader what you might do next once you have settled on an agreed definition.

 Overall, I found this article to be informative and a very useful practical approach to documenting and exploring the problem of reporting of trial recruitment. Some minor changes will enhance the quality and usefulness of the article and I look forward to reading about the next phase of this work.

Is the work clearly and accurately presented and does it cite the current literature?

Yes

If applicable, is the statistical analysis and its interpretation appropriate?

Not applicable

Are all the source data underlying the results available to ensure full reproducibility?

Yes

Is the study design appropriate and is the work technically sound?

Yes

Are the conclusions drawn adequately supported by the results?

Partly

Are sufficient details of methods and analysis provided to allow replication by others?

Yes

Reviewer Expertise:

Systematic reviews and evidence synthesis, evaluation (including trials).

I confirm that I have read this submission and believe that I have an appropriate level of expertise to confirm that it is of an acceptable scientific standard, however I have significant reservations, as outlined above.

# Response

**Published:** 2022-03-04

**Authors:** Valerie Smith

**Affiliations:** Trinity College Dublin and National University of Ireland Galway, Ireland

Many thanks for taking the time to consider our paper. We appreciate your valuable input and have replied to your comments, in sections below. Please see our amendments to the text in the revised paper. This revised version now also includes the findings and methods for Phase 2 of the Concept Analysis (and a final definition of ‘trial recruitment’).

**Abstract: **We now use the term ‘recruitment’ consistently in the abstract but note also that the term ‘recruitment’ is often referred to interchangeably in reports as ‘enrolment’. We have added a further sentence to the Background (Section 1.0) to explain this further.

**Background:** We have added a sentence to the Background to emphasize why variation in how recruitment is reported can be problematic.

**Methods:**

 In response to your comment on the exclusion of protocols: Thank you for this important point. We did consider trial protocols, however, we wished to analyse ‘actual’ recruitment data rather than what might be planned. We have clarified this with an added sentence to the paper (end of paragraph in ‘Dealing with meaning and measurement’ section).

 We have corrected Figure 3.

 We have moved the line "148 of the records reported on randomised trials and two reported on non-randomised trials" to the ‘Characteristics of included studies’ section.

 In response to your query about cluster, parallel and pragmatic RCT’s: Thank you for raising this important point, however, unfortunately we did not extract these specific types of data as our focus was on definition and description of recruitment irrespective of trial design. We do see, in hindsight, that this provides a limitation, and have noted this in the added text on limitations in in the Conclusion.

 We have revised the wording around proportions and ‘clearly identify’ in line with your suggestions.

**Conclusion:**

 We have now acknowledged the limitation that the type of randomised trial might also potentially influence the definition of recruitment in trial reports.

 In response to your comment about ‘enrolment’: We appreciate that the terms ‘enrolment’ and ‘recruitment’ are often used interchangeably and indeed ‘enrolment’ was a commonly used term in the analysed reports. As our aim was to develop a definition for ‘recruitment,’ which is not to say that ‘recruitment’ is a more useful term than ‘enrolment’ but the hope is that by providing clarity on at least one term, this will reduce ambiguity in trial reporting, and potentially encourage those reporting on trials to consistently use ‘recruitment’ based on this definition.

 In response to your comment about emphasising the importance of clear trial reporting and preliminary recommendations: We have added text to the end of the Conclusion which we hope addresses this point.

# Peer review: Reviewer response for version 1

**DOI:** 10.21956/hrbopenres.14299.r28598

**Published:** 2021-01-29

**Authors:** Hans Lund

**Affiliations:** 1 Section for Evidence-Based Practice, Western Norway University of Applied Sciences, Bergen, Norway

1. The authors state that "ambiguity in anchoring the trial start date, end date, recruitment and enrolment temporally to trial processes (e.g. invitation, consent, and randomisation) has the potential for variation in how recruitment is reported and understood in trial registries, trial protocols and trial reports." and thus in phase 1 aims to perform a concept analysis of ‘trial recruitment’ and develop a preliminary operational definition of ‘trial recruitment’. However, there is no systematic evaluation of earlier similar studies, and thus a limited justification of this study. The aim of the study makes it even more importantly to identify and consider earlier similar studies as the authors are looking for how a certain concept has been used and defined.
2. Whenever a concept is analyzed and discussed it would be helpful to identify, describe and use the existing theories about the concept. Having this theoretical background would be both helpful in analyzing the results of the study and helps the readers to evaluate the relevance of the interpretation of the data.
3. It may have been relevant to perform an analysis where the results randomized versus non-randomized included studies were performed.
4. All in all a very important and well-performed study.

Is the work clearly and accurately presented and does it cite the current literature?

Partly

If applicable, is the statistical analysis and its interpretation appropriate?

Yes

Are all the source data underlying the results available to ensure full reproducibility?

Yes

Is the study design appropriate and is the work technically sound?

Yes

Are the conclusions drawn adequately supported by the results?

Yes

Are sufficient details of methods and analysis provided to allow replication by others?

Yes

Reviewer Expertise:

Rehabilitation - Systematic Reviews - Evidence-Based Medicine - Evidence-Based Research

I confirm that I have read this submission and believe that I have an appropriate level of expertise to confirm that it is of an acceptable scientific standard, however I have significant reservations, as outlined above.

# Response

**Published:** 2022-03-04

**Authors:** Valerie Smith

**Affiliations:** Trinity College Dublin and National University of Ireland Galway, Ireland

Many thanks for taking the time to consider our paper. We appreciate your valuable input and have replied to your comments, numbered below. Please see our amendments to the text in the revised paper. This revised version now also includes the findings and methods for Phase 2 of the Concept Analysis (and a final definition of ‘trial recruitment’).

 1. We appreciate this important comment, thank you, however, we have not identified any previous similar studies including any previous concept analyses that have specifically analysed the concept of ‘trial recruitment’ and how it might be operationally defined. We have now emphasised this in the final paragraph of the Background section. This text is also expanded and further supported with revisions in line with comment 2 (see below).

 2. Similar to comment 1, we did not identify existing theories that explicitly analysed the concept of ‘trial recruitment’, however, we have now added text on previous studies that used theoretical frameworks in trying to understand or enhance trial recruitment strategies. This additional text and references (final paragraph of the Background section), we hope gives further justification for undertaking a formal concept analysis.

 3. We acknowledge that trial design could potentially impact on the variation and type of terminology used when reporting trials, for instance whether or not a trial is randomised and whether the trial includes a run-in period. We did not extract data relating to trial run-in periods and the majority of the trials analysed are randomised trials (n=148/150 are RCTs). We also refer to this issue in the limitations sections of the Conclusion.
